# Prevalence and genotypes of *Chlamydia psittaci* in pet birds of Hong Kong

**DOI:** 10.1371/journal.pone.0306528

**Published:** 2024-09-06

**Authors:** Jackie Cheuk Kei Ko, Yannes Wai Yan Choi, Emily Shui Kei Poon, Nicole Wyre, Jennifer Le Lin Go, Leo Lit Man Poon, Simon Yung Wa Sin

**Affiliations:** 1 School of Biological Sciences, The University of Hong Kong, Pok Fu Lam Road, Hong Kong, China; 2 Zodiac Pet & Exotic Hospital, Shop 101A to 103A, 1/F, Victoria Centre, 15 Watson Road, Fortress Hill, Hong Kong, China; 3 Centre for Comparative Medicine Research, Li Ka Shing Faculty of Medicine, The University of Hong Kong, Pok Fu Lam, Hong Kong, China; 4 School of Public Health, LKS Faculty of Medicine, The University of Hong Kong, Hong Kong, China; Huadong Research Institute for Medicine and Biotechniques, CHINA

## Abstract

Psittacosis, or parrot fever, is a zoonotic disease caused by *Chlamydia* species associated with birds. One of the causative agents of the disease is *Chlamydia psittaci*, which is commonly carried by psittacine and other bird species, can be highly pathogenic and virulent to humans. In Hong Kong, a city with high population density, psittacosis is a notifiable disease with over 60% of cases in the last decade resulting in hospitalization. However, the sources of transmission of *C*. *psittaci* and its prevalence in pet birds in Hong Kong are currently unknown. To evaluate the risks of psittacosis transmission through pet birds, we tested the presence of *C*. *psittaci* and determined its genotypes in samples obtained from 516 captive birds from households, pet shops, and a veterinary hospital in Hong Kong. Results revealed that five samples (0.97%), collected from budgerigars and cockatiels, were *C*. *psittaci*-positive, while four (80%) of them were obtained from pet shops. Our phylogenetic analysis revealed that all identified strains belonged to Genotype A and showed high similarity to other sequences of this genotype obtained from various geographical locations and host species, including mammals. Our findings provide evidence for the presence of *Chlamydia psittaci* and shed light on its sources in captive birds in Hong Kong. They highlight the potential zoonotic risks associated with this pathogen, which can affect both humans and wild birds.

## 1. Introduction

Psittacosis, also known as parrot fever, is a zoonotic disease caused by avian-associated *Chlamydia* species, with *Chlamydia psittaci* being the major and most studied causative agent [1]. In humans, *C*. *psittaci* infection can be fulminant or subclinical [2], leading to influenza-like illnesses, pneumonia, and even death, which is especially common in the elderly [3, 4]. Although antibiotic treatment for psittacosis is available, the disease remains an important health concern, especially for immunosuppressed individuals [3].

The host range of *C*. *psittaci* is broad, with over 465 bird species in 30 orders found to be vulnerable to its infection [5]. Although birds are the primary carriers, *C*. *psittaci* has also been detected in diverse animal species, including mammals such as dogs, cats, foxes, cattle, sheep, pigs, and horses, as well as reptiles such as crocodiles, lizards, and tortoises [6, 7]. In recent years, the reporting of *C*. *psittaci* infection in mammals, especially domestic animals, has become more frequent, raising concerns about zoonotic transmission to humans through these animals [6]. Preventing of zoonotic transmission to humans through contact in captive and domestic settings, such as pet keeping and agriculture, has been challenging as asymptomatic infections are common in most animal host species [8]. The emergence of chlamydial co-infections involving *C*. *psittaci* and other species, including *C*. *avium*, *C*. *abortus*, and *C*. *gallinacea* in pigeons [9, 10] and poultry such as ducks and chickens [11], as well as livestock animals such as cattle, pigs, and sheep [12, 13], further complicates the treatment and prevention of psittacosis. Mixed chlamydial infections in animals have been observed to exacerbate symptoms, including an increased chance of abortion [14]. Although co-infection of *C*. *avium*, *C*. *abortus*, or *C*. *gallinacea* with *C*. *psittaci* has not been recorded in humans, mixed *C*. *psittaci* and *C*. *pneumoniae* infection has been reported in a few human cases [15, 16]. These patients exhibited different levels of respiratory symptoms, from flu-like symptoms to dyspnea, pneumonia, and even global respiratory failure, as well as complications including myocarditis. Treatment of co-infections involves the use of a mixed combination of ceftriaxone and erythromycin or azithromycin [15, 16].

To date, 16 Genotypes (A, B, C, D, E, E/B, F, G, 6N, Mat116, M56, Daruma-1981, R54, 1V, WC, CPX0308) of *C*. *psittaci* have been identified based on sequences of the outer membrane protein A (*ompA*) gene [17]. The *ompA* gene has long served as a traditional marker for genotyping *C*. *psittaci* spp. Alongside other genotyping methods like MLST-typing and SNP-genotyping, *ompA* sequencing accurately reflects the phylogenetic relationships between *C*. *psittaci* strains, as demonstrated by whole genome sequences [18]. The differentiation of *ompA* genotypes primarily relies on the sequences of four variable domains (VDs), which encode motifs located in the outermost region of the expressed protein known as the Major Outer Membrane Protein (MOMP) [18, 19]. The *ompA* genotypes exhibit host tropism [20], potentially due to the role of MOMP in interacting with the host’s immune functions [21]. Genotype A predominantly associated with psittacine birds [5, 18, 22]. Genotype B is strictly linked to Columbiformes hosts [18], while other genotypes display a wide range of host preferences, including waterfowls, chickens, turkeys, pigeons, passerines, and other avian or mammalian species [8, 23]. Among all, Genotype A stands out as the most virulent and responsible for causing the majority of psittacosis outbreaks [18, 24]. Despite its strong association with parrot hosts, Genotype A strains possess the ability to infect several distantly related bird groups such as Columbiformes, and Passeriformes, as well as mammalian hosts such as cattle and sheep, suggesting the versatility of this Genotype [18, 25, 26]. Nonetheless, all *C*. *psittaci ompA* genotypes are capable of causing diseases and can be transmitted to humans [20].

*Chlamydia psittaci* has been identified as the causative agent of psittacosis in over 20 countries across four continents and is responsible for community-acquired pneumonia (CAP) in most of these countries [27]. Estimates suggest that up to 6.7% of all recorded cases of CAP could be attributed to *C*. *psittaci* [27]. While transmission from domestic fowls, including turkeys, chickens and ducks, have been reported, the majority of psittacosis cases involve transmission of *C*. *psittaci* from captive parrots to pet owners, breeders, or handlers [28]. Captive psittacine birds have historically been responsible for causing the most psittacosis outbreaks [6, 29] and are considered significant transmitters of psittacosis. However, despite psittacosis being an ongoing health issue, there is a notable lack of data regarding captive psittacines in Asia. In the past decade, the detection and characterization of *C*. *psittaci* in captive parrots have been limited to a few Asian countries or regions, such as Japan [30, 31] and China [32], with reported prevalence rates ranging from 3.1% to 20.7% [33]. Considering the potential danger posed by psittacine-carried strains in many parts of Asia [29, 34–37], it is crucial to enhance our understanding of the genotypes and transmission patterns of *C*. *psittaci* in pet parrots in Asia.

Noteworthily, psittacosis has been a persistent public health concern in Hong Kong, with at least ten cases reported annually in the past decade. It was designated as a notifiable infectious disease under the Prevention and Control of Disease Ordinance in 2018 [38]. Among recorded cases in the last decade, a quarter reported contact with pet birds, primarily parrots, or their droppings. Over 60% of patients were hospitalized, with two deaths occurring due to respiratory failure or pneumonia [38]. Despite the recurring cases and the high-density pet bird population in Hong Kong [39], there is a lack of information regarding the prevalence, source of transmission, and genotypes of *C*. *psittaci* in birds and humans. Assessing infection rates, distribution, and genotypes of *C*. *psittaci* in pet birds, especially parrots, and their owners is essential for understanding the epidemiology of the disease in the community.

The purpose of this study is to conduct a screening of *C*. *psittaci* in captive psittacine and passerine birds in Hong Kong. Our objective is to determine the prevalence and genotypes of the pathogen in captive birds from pet shops, households, and a veterinary clinic to gain more insights into its potential source of transmission. To achieve this, we developed a specific nested PCR assay targeting the *ompA* gene to detect *C*. *psittaci* DNA, and genotypes were identified through *ompA* sequencing. These findings will be valuable in preventing and managing psittacosis transmission in Hong Kong and expanding our knowledge about *C*. *psittaci* carried by pet birds in Asia.

## 2. Materials and methods

### 2.1 Sample collection

Between November 2019 and January 2022, a total of 516 fecal samples were collected (S1 Data). These samples were obtained from the cages of captive birds from 218 households (N = 346), 4 pet shops (N = 54), and a veterinary hospital (N = 116). The samples encompassed a wide range of popular pet bird species, including 43 psittacine species and 7 passerine species. Most samples were collected from individual birds, except for 17 samples from pet shops, which were collected from 4 cages housing multiple budgerigars or cockatiels. During the sampling process at the veterinary hospital, 8 of the birds were receiving antibiotics at the time. Among the antibiotics used were doxycycline, which had been added to the drinking water of a common hill myna (*Gracula reliqiosa*) a week prior to sampling, and enroflaxacin, which had been administered to treat a grey parrot (*Psittacus erithacus*), a peach-faced lovebird (*Agapornis roseicollis*), and a Pacific parrotlet (*Forpus coelestis*) [40–42]. Other antibiotics used were generally not effective against *C*. *psittaci*, including chloramphenicol, amoxicillin-clavulanic acid, and metronidazole. All samples were stored in 100% ethanol at -20°C shortly after collection to preserve DNA in the samples. Whenever possible, information on the age, sex, symptoms, and medical history of each sampled bird, was obtained. This study received approval from the Human Research Ethics Committee (EA1912038) and Animal Research Ethics Committee (5264–19) of the University of Hong Kong; as well as the Department of Health [(19–1499) in DH/HT&A/8/2/3 Pt. 3] of the HKSAR Government.

### 2.2 DNA extraction

For DNA extraction from bird fecal samples, the E.Z.N.A. Stool DNA Kit (Omega Bio-tek, Norcross, USA) was used. Approximately 200 mg of each sample was used for extraction following the manufacturer’s protocol. Sample homogenization was achieved using a TissueLyser II (Qiagen, Hilden, Germany) with 5mm stainless steel beads (Qiagen). The eluted DNA was stored in 30–50μL of elution buffer at -20°C.

#### 2.3 Nested PCR assays for detecting *C*. *psittaci*

Nested PCR assays were developed to detect *C*. *psittaci* DNA in bird fecal samples. Using the *ompA* sequences of *C*. *psittaci*, *Chlamydia trachomatis*, and *Chlamydia pneumonia* retrieved from GenBank, two pairs of primers were designed to amplify two different regions of the *ompA* gene of *C*. *psittaci*, respectively (S2 Data). Primer pairs O1 and N1 were used to amplify the variable domain (VD) I, while pairs O2 and N2 were used to amplify VD III-IV.

In each reaction for the first PCR, 5μL of extracted DNA was used as template in a total volume of 25μL, with primer concentration of 0.6 μM (IDT, Coralville, USA). On the other hand, reaction mixture for the nested PCR consisted of 1μL of amplified first PCR product as template in a total volume of 25μL, with primer concentration of 0.6 μM (IDT). Touchdown conditions were used for the first PCR, while conventional PCR condition was used for nested reactions. The temperature condition for the first PCR was 95°C for 2 min, followed by 40 cycles of 95°C for 30 sec, 63.5–59.5°C (63.5–60.5°C for the first 4 cycles and 59.5°C for the remaining 36 cycles) for 30 sec, 72°C for 45 sec, and 72°C for 5 min. For the nested PCR, the condition was 95°C for 2 min, followed by 40 cycles of 95°C for 30 sec, 59.5°C for 30 sec, 72°C for 45 sec, and 72°C for 5 min.

The study employed Amplirun *C*. *psittaci* DNA Control (Vircell Microbiologists, Granada, Spain) as a ten-fold diluted positive control and UltraPure water (Invitrogen) as a negative control in the initial PCR reactions. To avoid potential cross-contamination between samples, 28 samples were tested per batch, along with one positive and one negative control. In total, 30 rounds of detection PCR were conducted to analyze all samples. The expected results were obtained in all positive and negative control reactions, which were confirmed by DNA gel analysis. All PCR products with expected size were sequenced by BGI (BGI Genomics, Hong Kong), and the sequences were analyzed using Geneious Prime 8.1.9. The identities of sequences were verified by performing searches in the basic local alignment search tool (BLAST) against GenBank (NCBI) database.

### 2.4 The *ompA* gene amplification for genotype identification

The *ompA* gene amplification was performed to identify genotypes of *C*. *psittaci* in positive samples. Initially, the protocol described by Madani et al. was attempted but yielded suboptimal amplification [43]. Therefore, three pairs of primers were designed based on *C*. *psittaci ompA* sequences from GenBank, to amplify three overlapping regions within the gene, to obtain the full *ompA* sequences (S3 Data). Primer pairs C2 and C3 were used for VD I-II and VD III-IV amplification, respectively, while primer pair C3 was used to amplify VD I-IV. The reaction mixtures consisted of 5μL of extract-ed DNA as a template in a total volume of 40μL, with primer concentration of 0.4μM (IDT). Touchdown conditions were used for the reaction. For reaction C1, the temperature condition was 95°C for 2 min, followed by 40 cycles of 95°C for 30 sec, 62.5–60.5°C (62.5–61.5°C for the first 2 cycles, and 60.5°C for the remaining 38 cycles) for 30 sec, 72°C for 45 sec, and 72°C for 5 min. For reaction C2, temperature condition was 95°C for 2 min, followed by 40 cycles of 95°C for 30 sec, 59–57°C (59–58°C for the first 2 cycles, and 57°C for the remaining 38 cycles) for 30 sec, 72°C for 45 sec, and 72°C for 5 min. For re-action C3, temperature condition was 95°C for 2 min, followed by 40 cycles of 95°C for 30 sec, 60.5–52.5°C (60.5–53.5°C for the first 8 cycles, and 52.5°C for the remaining 32 cycles) for 30 sec, 72°C for 45 sec, and 72°C for 5 min. Positive and negative controls were included using diluted *C*. *psittaci* DNA control and UltraPure DNase/RNase-free distilled water (Invitrogen), respectively. PCR products were sequenced and analyzed using Geneious Prime 8.1.9, with sequence identities verified through BLAST searches against GenBank (NCBI) database. Sequences ranging from 1111 to 1128bp were obtained from four positive samples (GenBank accession numbers: OP594252, OP594253, OP594255, OP594256). Due to subpar sequencing results, the remaining positive sample (OP594254) missed a region of approximately 323bp, spanning through VD2 and VD3.

### 2.5 Statistical analyses

Confidence intervals of *C*. *psittaci* prevalences in each sampled species were calculated using the Reiczigel method [44]. Fischer’s’ exact test was subsequently employed to test for significant differences between prevalences between sources and host species.

### 2.6 Genotype determination and phylogenetic relationship reconstruction

We calculated pairwise genetic distances using *ompA* sequences amplified from positive samples, as well as deposited sequences from the Public databases for molecular typing and microbial genome diversity (PubMLST) and GenBank databases [45]. Subsequently, we reconstructed a phylogenetic tree.

Initially, *ompA* sequences were extracted from all deposited *C*. *psittaci* genomes in the PubMLST database. These retrieved sequences, along with all *ompA* entries from GenBank, were aligned with the sample sequences using MAFFT. Based on sequence homology, as well as country of origin, host species, and sampling time of the retrieved sequences, 68 representative sequences were selected for genetic distance calculation and phylogenetic tree reconstruction alongside the five sample sequences (S4 Data). Additionally, certain redundant sequences with unique host species and country-of-origin combinations were included. In cases where redundant sequences shared the same host species and country of origin, the most recently discovered sequence was chosen.

We used Geneious Prime 8.1.9 to calculate nucleotide p-distances. Model selection and phylogenetic tree construction were conducted using IQ-Tree [46–50]. A maximum likelihood (ML) tree was constructed with 1000 bootstrap replicates. The HKY+F+G4 model (Hasegawa-Kishino-Yano model, which considers empirical base frequencies and applies gamma rate heterogeneity) was selected (Hasegawa et al. 1985). The phylogeny was visualized and modified using the interactive Tree of Life (iTOL v6) [51].

## 3. Results

### 3.1 Prevalence of *C*. *psittaci* in sampled birds

Out of 516 bird fecal samples, five (0.97%) were detected positive for *C*. *psittaci* (Table 1). The positive samples were collected from two parrot species, with four from budgerigars (*Melopsittacus undulatus*; 13.8%) and one from cockatiels (*Nymphicus hollandicus*; 1.61%). Fischer’s exact test did not reveal a significant difference in *C*. *psittaci* prevalences between host species (*P* = 0.32). One of the positive samples was collected from a singly housed budgerigar from a household, while the other three were collected from a cage containing 10 budgerigars in a pet shop. According to the pet owner, the budgerigar from the household did not exhibit any observable symptoms. It was uncertain which individual, or how many individuals, were infected with *C*. *psittaci* in the cage of the positive budgerigars from the pet shop, as two negative samples were collected from the same cage at the same time. At least one bird in that cage was likely uninfected. None of the budgerigars in the sampled cage showed any observable symptoms during the time of sample collection. The positive sample from the cockatiels was collected from a cage housing seven birds of the same species in the same pet shop as the budgerigars, and two other samples collected from the same cage were negative for *C*. *psittaci*. Of the seven cockatiels in the same cage, one had observable feather loss, primarily from the crown. Moreover, the collected sample was watery and appeared diarrheic.

**Table 1:** *psittaci* in bird samples from households, pet shops, and a veterinary hospital. **Summary of the prevalences of *C*.** Confidence intervals (CI) were calculated using the Reiczigel method [44].

Common name.	Species name	Total no. of samples (%; 95% CI)	Total no. of samples from households	Total no. of samples from pet shops	Total no. of samples from an animal hospital
**Budgerigar**	*Melopsittacus undulatus*	4/29 (13.79%; 1.01–30.50%)	1/13 (7.69%)	3/11 (27.27%)	0/5
**Cockatiel**	*Nymphicus hollandicus*	1/62 (1.61%; 0.00–8.88%)	0/39	1/6 (16.67%)	0/17
**Total number of *C*. *psittaci*-positive samples from parrots**		5/492 (1.02%; 0.00–3.81%)	1/339 (0.29%)	4/39 (10.26%)	0/114
**Total number of *C*. *psittaci*-positive samples from all bird species**		5/516 (0.97%; 0.12–3.74%)	1/346 (0.29%; 0.00–3.23%)	4/54 (7.41%; 0.00–17.21%)	0/116

The prevalence of *C*. *psittaci* in samples collected from pet shops was found to be 7.41%, which was much higher compared to the prevalence in households (0.29%) and the animal clinic (0.00%). The differences in prevalences among these sources were statistically significant as tested using Fischer’s exact test (*P*<0.001).

### 3.2 The *ompA* sequence characterization and analysis

Pairwise distances revealed high similarity among all amplified *ompA* sequences, with nucleotide distances ranging from 91.3% to 92.5% (S5 Data) between OP594252 (referred to as 252), OP594253 (253), OP594255 (255), and OP594256 (256). However, for OP594254 (254) that missed a region in VD2 and VD3, the percentage similarities with other sequences were 70.8% to 72.2%. When excluding those missing regions, sequences 254 (sampled from budgerigar in the pet shop), 255 (from a cockatiel in the pet shop), and 256 (from a budgerigar in the household) were found to be identical. The other two sequences differed from each other by unique single nucleotide polymorphisms (SNPs). Sequences 252 and 253 had point deletions at positions 89 and 122 of the gene, respectively, when aligned with complete *ompA* sequences from reference strains. These deletions were located in the conserved region in front of VD1. Additionally, sequence 253 demonstrated a substitution at position 1214 (T → C), which is situated at the conserved region following VD4.

When comparing sampled *C*. *psittaci* with retrieved *ompA* sequences from the MLST and GenBank databases, nucleotide distances revealed high similarities with sequences of Genotypes A, B, E, and E/B (S6 Data). All sequences, except 254, showed over 90% similarities with sequences of Genotype A (90.9–92.8%), B (90.3–92,1%), and E (90.8–92%), as well as 89–90.6% similarity with sequences of Genotype Mat116. Sequences 254–256 were found to be identical to reference strains 84/55 (CP003790) and Cal-10 (MLST id: 882) of Genotype A. Sequences 254–256 are characterized by 2 major SNPs at positions 355 (C → G) and 533 (T → C) when compared to the standard reference strain 6BC. The former SNP was located at the inter-domain region between VD1 and VD2, while the latter was located near the end of VD2. Both SNPs resulted in non-synonymous substitutions (former: Gln → Glu; latter: Met → Thr). Sequence 253 had a substitution (T → C) at position 1214, as described earlier, which was not observed in any of the reference strains (6BC, 84/55, or Cal-10) of Genotype A. When compared to the mentioned reference strains, the substitution resulted in a synonymous mutation (AAT → AAC).

### 3.3 Phylogenetic analysis

**Fig 1:**
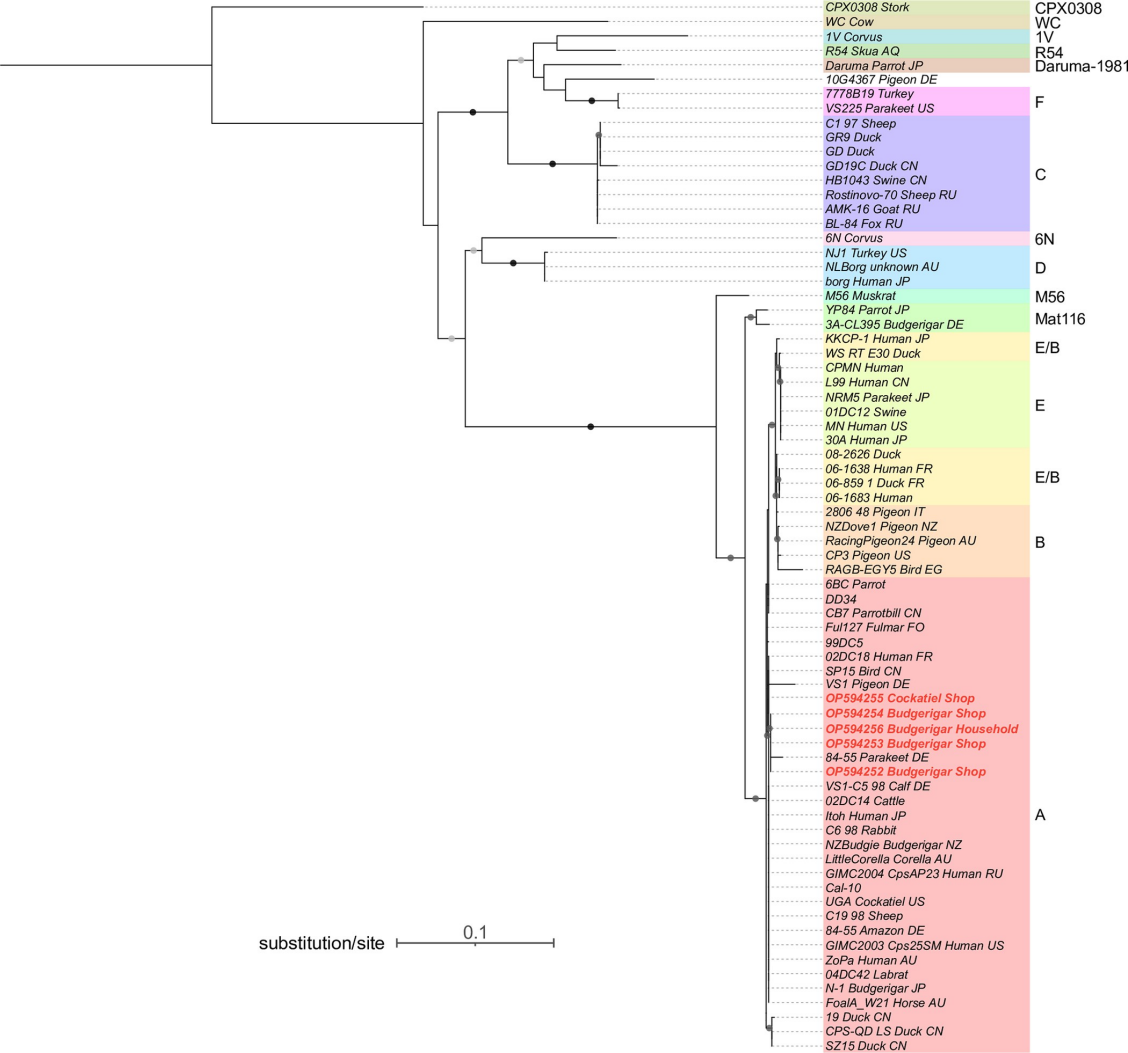
Phylogenetic tree constructed using *Chlamydia psittaci ompA* gene sequences. The tree includes sequences obtained from positive samples in this study (shaded in light red) as well as sequences retrieved from PubMLST and GenBank [45]. Sequences discovered in this study are shown in red font. Node labels consist of strain names followed by the host species and the sampled country, whenever available. Bootstrap values of 60 or above are presented as round shapes on the branches. Light grey shapes indicate bootstrap values above 60 and below 79, dark grey shapes indicate values above 80 and below 100, while black shapes represent a value of 100.

The ML tree obtained from the analysis demonstrated distinct and well-supported clustering of most genotypes, although Genotype E/B showed some ambiguity (Fig 1). The Genotype E/B sequences were separated into two clades, one clustering with Genotype B sequences and the other clustering with Genotype E sequences. However, it was observed that the majority of E/B sequences showed closer relatedness to each other, with the exception of 08–2626_Duck (E/B), which appeared to be closer to the Genotype B sequences.

The *ompA* sequences identified in this study were found to belong to the same clade as many other Genotype A sequences, including the reference strains 84/55 and Cal-10. The short branch lengths observed within this clade suggest a low variation for this Genotype at the *ompA* gene. Sequences 253, 254, 256, and 252, which were all sampled from budgerigars, formed a highly supported monophyletic clade, along with an 84/55 strain that was isolated from a parakeet (Y16561). In contrast, sequence 255, obtained from a cage of cockatiel, was found to be more closely related to multiple other Genotype A strains, including Cal-10, VS1, and an 84/55 strain that was obtained from an Amazon parrot. It is noteworthy that these Genotype A strains were collected from a diverse range of host species, including pigeon, vinous-throated parrotbill, rabbit, sheep, cattle, lab rat, and human, and were obtained from diverse geographical locations, including China, Japan, Germany, Russia, Australia, and the United States (US).

## 4. Discussion

Our study conducted the first screening of *C*. *psittaci* in local captive birds in Hong Kong. Despite the high-density pet bird population and active pet bird trade market [39, 52, 53], the prevalence of *C*. *psittaci* (i.e., 0.97%) was lower than that of most nearby regions, including Yunnan province, Gansu province, Beijing city, and Weifang city of China. These regions reported *C*. *psittaci* prevalences ranging from 10.8% to 35.57% in pet birds from pet markets, zoos, or unknown sources during 2015–2016 [32, 54, 55]. In Taiwan, a prevalence of 3.1% was reported in breeding facilities, a bird imports corporate, a zoo, and a veterinary hospital in 2019 [23].

Among the 516 samples obtained from 50 psittacine and passerine species from households, pet shops and an animal clinic, positive samples were found in budgerigars and cockatiels, which are popular pet parrots both locally and globally. High prevalences of *C*. *psittaci* in these two parrot species have been reported in multiple regions [4, 20, 34, 37, 56], with higher prevalence rates compared to other parrots [57]. Notably, we observed low prevalences in several heavily traded parrot species, including peach-faced lovebirds, grey parrots, monk parakeets (*Myiopsitta monachus*), turquoise-fronted amazons (*Amazona aestiva*), and others. Particularly, despite sampling over 10 individuals from pet shops, we found zero prevalence in peach-faced lovebirds, which contradicts the high infection rates reported in previous studies [32, 37, 58, 59].

Among the three sample sources, the number of positive samples collected from pet shops was significantly more than the other sources (households or the animal clinic). This finding aligns with previous studies that have identified pet shops or breeding facilities as the primary sources of *C*. *psittaci* transmission [4, 8, 32]. Risk factors associated with pet shops, such as poor hygiene conditions, high bird density, or increased stress levels, can contribute to the proliferation and transmission of *C*. *psittaci* [5]. Moreover, most of the positive parrots did not exhibit any observable symptoms, which can make it easier for handlers and pet owners to overlook the potential risks of contracting *C*. *psittaci* from these birds.

To determine the genotype of *C*. *psittaci* present in the positive samples, we used a majority of *ompA* sequences available in the PubMLST and GenBank databases to compute pairwise genetic distances and construct a phylogenetic tree with the amplified sequences. This method was employed to increase the accuracy of genotype and phylogenetic relationship identification. However, no definitive conclusions could be drawn based on pairwise distances, as our sequences exhibited high similarity with multiple genotypes, including Genotypes A, B, E, and Mat116. Therefore, genotype determination was based on phylogeny, which revealed that our amplified sequences were closely related to Genotype A sequences. *C*. *psittaci* Genotype A strains have a strong association with psittacine hosts, but they are also capable of infecting other species, such as birds, rodents, livestock animals, and humans [18, 25, 26]. This genotype also contains the greatest number of virulent strains, making it a significant concern for zoonotic transmission [18, 24]. Within the Genotype A strains, the *C*. *psittaci* found in our samples were more closely related to reference strains 84/55, VS1, and Cal-1, rather than to the typical 6BC strain that is considered the most virulent *C*. *psittaci* strain [24, 60, 61]. The short branch lengths observed throughout the Genotype A clade suggested a low variation in the *ompA* sequences within this Genotype. This finding is consistent with the previous conclusion by Read et al. [60], who used whole genome sequences to suggest the recent emergence of the 6BC lineage [60]. Notably, our phylogenetic tree revealed that this clade consisted of several strains with closely related, if not identical, *ompA* sequences that were sampled from various countries or regions, including some from different continents. These countries include Australia, New Zealand, Germany, France, Russia, the US, and even the Faroe Islands. This observation suggests a recent and rapid global expansion of *C*. *psittaci* Genotype A.

All five sequences belonged to the same monophyletic clade along with multiple other Genotype A sequences, however, there was an unexpected clustering pattern within this clade. Four sequences, all derived from budgerigars, formed a subclade together with the sequence of an 84/55 strain isolated from a parakeet. Conversely, the remaining sequence from cockatiel showed closer relatedness to various other Genotype A strains. This outcome was surprising as these sequences were collected from the same pet shop and were expected to cluster closely together. The distinct clustering pattern, with the only more distantly related sequence originating from a different host species, suggests the possibility of host-dependent divergence among the identified *C*. *psittaci* strains within Genotype A. It may also indicate the potential for adaptive evolution within the Genotype A *ompA* gene, potentially leading to specialization within the budgerigar host. However, these speculations require further verification with additional evidence.

The 84/55 strain, which clustered together with our amplified sequences, was originally isolated from a parakeet in the study conducted by Vanrompay et al. in 1998 [62]. In the article, it was mentioned that the strain was obtained from a veterinary hospital in Germany, cultured, and purified specifically for *ompA* sequencing and cloning purposes. However, the study did not provide detailed clinical information regarding the strain, such as its virulence in different hosts, including the parakeet and the transfected turkey. Referring to the nucleotide sequences of the diverged sequences, these sequences differentiate themselves from the other sequences mainly by point deletions within the first conserved domain. Although these deletions are supported by high-quality chromatogram data (S5 and S7 Data), it remains uncertain whether they are the results of amplification errors, such as PCR errors or primer biases [63, 64]. To verify the authenticity of these deletions, it is necessary to amplify the original samples using different primer combinations to achieve broader coverage at the same site. Additionally, employing genotyping methods like MLST and SNP-genotyping [18, 65], which rely on multiple genetic signatures for membership establishments, would serve as valuable alternatives to *ompA* sequencing. Implementing these methods would enhance the resolution of strain identification and aid in verifying the authenticity of the observed phylogenetic pattern.

The close relationship between the sampled *C*. *psittaci* strains and Genotype A strains, known for their ability to infect a wide range of hosts, suggests that these strains likely have the potential to infect humans and other bird groups [66]. Isolates within the Genotype A clade have been found in various hosts, including psittacine birds, passerines (such as parrotbills), ducks, fulmars, livestock animals like cattle, sheep, and horses, rodents like rabbits and lab mice, as well as humans. As mentioned in Sachse et al.’s recent study [18] and supported by Hogerwerf et al. [6], psittacine birds are the primary hosts for this genotype, and the fact that Genotype A strains have been isolated from diverse mammalian hosts suggests that these animals were initially infected by birds. Consequently, pet owners and handlers who come into contact with birds carrying these identified strains are at high risk of contracting psittacosis. Moreover, although we did not detect *C*. *psittaci* in passerine birds, it is likely that the identified strains can infect other bird species, potentially acting as reservoirs [67].

Given that most pet shops are open-air and frequented by feral birds, such as pigeons and sparrows, which come into close proximity with bird cages for food and water, there is an elevated risk of psittacosis. This increases the possibility of pathogen spillage into wild bird populations, posing a threat to bird species of conservation concern, such as the critically endangered yellow-crested cockatoo (*Cacatua sulphurea*) found in urban areas of Hong Kong [68]. The spillover of Chlamydiaceae, along with other avian pathogens, has recently been identified as an emerging zoonotic threat to humans and endemic bird species in Australia due to their high genetic diversity [69]. Infected feral birds, acting as reservoirs, can serve as long-distance transmission vectors of *C*. *psittaci* to distant birds and humans, even without direct contact. In urban cities like Hong Kong, where humans coexist closely with urban wildlife, pathogens can be transmitted through the air via aerosols from bird feces and feather dust [70], further heightening concerns regarding the zoonotic transmission of avian pathogens. While more than half of past psittacosis patients did not have direct contact with birds [38], we suggest that surveys of *C*. *psittaci*, and possibly other *Chlamydia* species, shall be extended to feral bird populations in Hong Kong to investigate their role in psittacosis transmission. Simultaneously, genotyping of *C*. *psittaci* strains in psittacosis patients is crucial to understand the specific genotypes and strains responsible for the spread of this disease. Genotyping methods with higher resolution, such as MLST genotyping and SNP-typing, will be valuable in further elucidating the transmission patterns of this pathogen within the city.

In addition to *C*. *psittaci*, there has been a rise psittacosis cases worldwide caused by other *Chlamydia* species, especially *C*. *avium* and *C*. *abortus*, which are also carried by pet birds [23, 71]. Although limited reports have described these *Chlamydia* species in parrot hosts within Asia [30], it is crucial to conduct future research on pan-*Chlamydia* detection in both pet birds and wild birds in Hong Kong and other parts of Asia to understand their roles in causing psittacosis in regions where the disease is a notifiable problem. Pan-Chlamydial detection is especially important as co-infections of the aforementioned closely related species with *C*. *psittaci* have become increasingly prevalent in various avian species, such as pigeons [9, 10, 72]. The impact of co-infection with these bird-associated chlamydia species on elevated symptoms or complications in humans remains uncertain and requires more investigation.

Our study has a few limitations. Firstly, we only tested fecal samples, which may have resulted in a biased detection rate compared to other sampling sites. It has been suggested that fecal samples, along with cloacal samples, may have a lower positivity rate than pharyngeal swab samples [73]. Secondly, despite the nested PCR approach being widely recognized and supported in previous literature [2, 43, 74], it may have limitations in sensitivity when compared to alternative detection methods such as qPCR and microarray-based approaches. Therefore, our findings may not be directly comparable to those of other detection methods and could potentially include false negative results. Thus, we encourage further investigations using methods with higher sensitivity, as described earlier, to gain a more comprehensive understanding of the prevalence and major sources *C*. *psittaci* in bird populations in Hong Kong. Additionally, since this study did not include analyses of *C*. *psittaci* infectivity and vitality analyses, it remains unknown whether the detected *C*. *psittaci* DNA reflects the presence of live, transmittable bacteria. Without this information, it is difficult to evaluate the clinical and epidemiological implications of the observed *C*. *psittaci* DNA positivity. Further research is necessary to elucidate the transmission dynamics and clinical significance of *C*. *psittaci* infections in parrots in Hong Kong.

## 5. Conclusions

The study aimed to investigate the prevalence and genotypes of *C*. *psittaci* among pet birds in Hong Kong, including those from households, pet shops, and a veterinary clinic. Out of the 516 samples tested, *C*. *psittaci* DNA was detected in 5 cases (0.97%). All identified *C*. *psittaci ompA* sequences belong to Genotype A and showed a close resemblance to reference strains 84/55, VS1, and Cal-10. Notably, a significant number of positive samples were obtained from parrots in pet shops, suggesting the potential for widespread transmission of *C*. *psittaci* in pet birds through trade across the country. To better understand the modes of transmission of psittacosis to humans, our report highlights the importance of detecting and genotyping *C*. *psittaci* strains in feral birds and psittacosis patients at higher resolution, as well as the need for future pan-*Chlamydia* detection in potential animal hosts.

## Supporting information

S1 Data (XLSX)

S2 Data (XLSX)

S3 Data (XLSX)

S4 Data (XLSX)

S5 Data (AB1)

S6 Data (XLSX)

S7 Data (AB1)
